# MicroRNA-34a is a tumor suppressor in choriocarcinoma via regulation of Delta-like1

**DOI:** 10.1186/1471-2407-13-25

**Published:** 2013-01-18

**Authors:** Ronald TK Pang, Carmen ON Leung, Cheuk-Lun Lee, Kevin KW Lam, Tian-Min Ye, Philip CN Chiu, William SB Yeung

**Affiliations:** 1 Department of Obstetrics and Gynaecology, The University of Hong Kong, Pokfulam Road, Hong Kong, China; 2 Center for Reproduction, Development and Growth, The University of Hong Kong, Pokfulam Road, Hong Kong, China

**Keywords:** miR-34a, DLL1, Choriocarcinoma, Invasion, Notch

## Abstract

**Background:**

Choriocarcinoma is a gestational trophoblastic tumor which causes high mortality if left untreated. MicroRNAs (miRNAs) are small non protein-coding RNAs which inhibit target gene expression. The role of miRNAs in choriocarcinoma, however, is not well understood. In this study, we examined the effect of miR-34a in choriocarcinoma.

**Methods:**

MiR-34a was either inhibited or ectopically expressed transiently in two choriocarcinoma cell lines (BeWo and JEG-3) respectively. Its actions on cell invasion, proliferation and colony formation at low cell density were examined. The miR-34a putative target Notch ligand Delta-like 1 (DLL1) was identified by adoption of different approaches including: *in-silico* analysis, functional luciferase assay and western blotting. Real-time quantitative polymerase chain reaction was used to quantify changes in the expression of matrix proteinase in the treated cells. To nullify the effect of miR-34a ectopic expression, we activated Notch signaling through force-expression of the Notch intracellular domain in the miR-34a force-expressed cells. In addition, we studied the importance of DLL1 in BeWo cell invasion through ligand stimulation and antibody inhibition. Furthermore, the induction in tumor formation of miR-34a-inhibited BeWo cells in SCID mice was investigated.

**Results:**

Transient miR-34a force-expression significantly suppressed cell proliferation and invasion in BeWo and JEG-3 cells. *In silicon* miRNA target prediction, luciferase functional assays and Western blotting analysis demonstrated that miR-34a regulated DLL1 expression in both cell lines. Although force-expression of miR-34a suppressed the expression of DLL1 and NOTCH1, the extent of suppression was higher in DLL1 than NOTCH1 in both cell lines. MiR-34a-mediated DLL1 suppression led to reduced matrix metallopeptidase 9 and urokinase-type plasminogen activator expression. The effect of miR-34a on cell invasion was partially nullified by Notch signaling activation. DLL1 ligand stimulated while anti-DLL1 antibody treatment suppressed cell invasion. Mice inoculated with BeWo cells transfected with miR-34a inhibitor had significantly larger xenografts and stronger DLL1 expression than those with cells transfected with the control inhibitor.

**Conclusions:**

MiR-34a reduced cell proliferation and invasiveness, at least, partially through its inhibitory effect on DLL1.

## Background

Choriocarcinoma is a highly malignant trophoblastic tumor characterized by abnormal trophoblastic hyperplasia and anaplasia. It can be derived either from a normal or pathological pregnancy like molar pregnancies, induced/spontaneous abortions, ectopic pregnancies and preterm deliveries [1]. Although choriocarcinoma is a rare disease, if left untreated, can spread rapidly and has a mortality rate of nearly 100% [2]. During organ transplantation, dissemination of choriocarcinoma cells from donors to recipients can lead to quick death of the recipients [3]. Our knowledge on choriocarcinoma is very limited due to its rarity and lack of proper controls in studies. Besides, heterogeneous causes of the disease make study of the disease much more complicated; cytogenetic analyses indicate that nearly all chromosomes can be affected and no consistent abnormality has been identified in choriocarcinoma [4].

MicroRNAs (miRNAs) are small untranslated RNAs that inhibit expression of target genes through translational inhibition or transcriptional silencing [5]. Bioinformatics analysis predicts that 30% of all the protein-coding genes are targets of miRNAs [6]. MiRNAs is involved in various physiological processes while aberrant miRNA expressions are usually pathological. Previously, only the roles of miR-141 and miR-199b in choriocarcinoma were reported [7,8]. The significance of other miRNAs in choriocarcinoma is not known.

The miR-34 family members share high sequence homology [9]. Among these, miR-34a is one of the earliest known miRNA tumor suppressor and is directly transactivated by p53 [10,11]. In this study, we used BeWo and JEG-3 cells as model to examine the role of miR-34a as a tumor-suppressor in choriocarcinoma. These 2 cell lines are widely used for the study of trophoblast physiology and trophoblastic cancer. Hence, we used gain/loss of function approach and demonstrated that miR-34a affected proliferation, colony-formation and invasion of choriocarcinoma cells *in vitro* and the tumor formation capability *in vivo*.

Notch signaling is a short range communication transducer system which is important in many physiological and pathological conditions [12] and is highly conserved. There are 4 Notch receptors (Notch 1–4) and 5 Notch ligands (DLL1, 3, 4 and Jagged1, 2) and belongs to the type I membrane-bound proteins. Upon ligand binding, the intracellular domain of the Notch receptor (NCID) is cleaved and translocated into the nucleus, where it acts as a transcriptional factor for target gene activation [13]. Bioinformatics analyses suggest that the Notch ligand, delta-like one (DLL1) is a target of miR-34a. This was further confirmed in the present study by the 3’-untranslated region (UTR) luciferase functional assay. The data also demonstrated that DLL1 and Notch signaling mediated the action of miR-34a in cell invasion.

## Methods

### Cell culture

The BeWo cells and JEG-3 cells (American Type Culture Collection, Manassas, VA) were cultured respectively in F12K medium or DMEM medium, (Invitrogen, Carlsad, CA) supplemented with 10% fetal bovine serum (FBS), 50 U/ml of penicillin and 50 μg/ml of streptomycin (Invitrogen). For force-expression of miR-34a, 1 × 10^5^ cells were seeded in 12-well culture plates 1 day before transfection either with 50 nM of precursor of miR-34a (pre-miR-34a) or pre-miR-Scramble (Negative Control #1, Ambion, Austin, TX) by Lipofectamine 2000 (Invitrogen). For activation of Notch signaling, a Notch NCID expression plasmid (pCDNA6-Notch NCID, a kind gift from Prof. Jon Aster, Brigham and Women’s Hospital and Harvard Medical School, Boston, Massachusetts, USA) was used. In control experiments, the cells were transfected with an empty vector (pCDNA6).

### Proliferation assay

Cell proliferation was estimated by the CyQuant® cell proliferation assay (Invitrogen) according to the manufacturer’s protocol. Fluorescence signal with excitation at 485 nm and emission at 530 nm was measured by a microplate reader (Tecan Group Ltd, Männedorf, Switzerland).

### Invasion assay

We used the BD Matrigel Invasion Chamber (8-μm pore size; BD Biosciences, Franklin Lakes, NJ) to quantify cell invasion. The transfected cells in FBS-free culture medium were seeded onto the upper chamber while the lower chamber was filled with normal FBS-containing medium. For DLL1 stimulation, 2.5 μg of recombinant DLL1 (R&D systems, Minneapolis, MN) was added to the upper chamber. In the control experiment, the same volume of DMSO was added to the cells. For antibody inhibition, 5 μg of polyclonal anti-DLL1 antibody (Santa Cruz Biotechnology, Santa Cruz, CA) was added to the upper chamber during seeding, and fresh antibody was added every 24 hours. After 48 hours, the cells remained in the upper chamber were removed by cotton swabs, whilst those that had invaded through the matrix between the two chambers were visualized by staining with 0.1% of crystal violet (Sigma-Aldrich, St Louis, MO). To quantify the invasion result, the dye was dissolved in 10% acetic acid and the absorbance was measured by a microplate reader. Parallel experiments on cell proliferation were performed to estimate the effect of cell proliferation on the results of cell invasion.

### Total RNA extraction, reverse transcription and quantitative real-time quantitative PCRs (RT-qPCR)

Total RNA was prepared by using the *mir*Vana^TM^ miRNA Isolation Kit (Ambion) according to the manufacturer’s protocol. For assaying mRNA, first-strand cDNA was synthesized by the High Capacity cDNA Reverse Transcription kit (Applied Biosystems, Foster City, CA) and the target gene expression was quantified by the TaqMan® Gene Expression Assays (Applied Biosystems) using an Applied Biosystems 7500 Detection system (Applied Biosystems). The expression of mRNA was determined from the threshold cycle (Ct), and the relative expression levels were calculated by the 2^-ΔΔCt^ method [14]. The relative expression levels were normalized with the expression of 18S mRNA. For measuring miRNAs, the first-strand cDNA was synthesized by the TaqMan® MicroRNA Reverse Transcription kit (Applied Biosystems) and the miRNA expression was quantified by the TaqMan® MicroRNA assay (Applied Biosystems). The relative expression of miR-34a was calculated as the above and the levels were normalized with the expression of the small RNA RNU6B.

### 3’UTR functional luciferase assays

Oligonucleotides were synthesized according to the nucleotide sequence of potential miR-34a binding regions identified by TargetScan5.2 on DLL1 (355–361 of DLL1 3’UTR, NCBI reference sequence: NM_005618.3). Specific primers were purchased from Invitrogen (Forward: 5’-TCCTCGAGAA TTAGAAACAC AAACACTGCC TGCGGCCGCT G-3’ and Reverse: 5’-CAGCGGCCGC AGGCAGTGTT TGTGTTTCTA ATTCTCGAGG A-3’). The DNA fragment was cloned into the Xho I and Not I sites of the pSiCheck™-2vector (Promega, Madison, WI). The vector was transfected with either pre-miR-34a or pre-Scramble into the cells (Ambion). At 48-hour post-transfection, the cells were lysed and the luciferase activities in the lysate were measured by the Dual Luciferase Reporter Assay System (Promega). The effect of the miRNA was measured by the activity of the Renilla luciferase normalized to that of the firefly luciferase. To test the specificity of the interaction between miR-34a and 3’UTR of DLL1, the miR-34a seed binding region on the 3’UTR of DLL1 was mutated. The mutant construct was generated with specific primers (Forward: 5’-TCCTCGAGAA TTAGAAACAC AAAGAGTACT TGCGGCCGCT G-3’ and Reverse: 5’-CAGCGGCCGC AAGTACTCTT TGTGTTTCTA ATTCTCGAGG A-3’; underlined regions denote the mutated sequences) and cloned into the pSiCheck™-2vector as described above.

### Colony formation assay

BeWo and JEG-3 cells transfected with pre-miR-34a or pre-Scramble were seeded at a density of 20 cells/cm^2^ in normal culture medium as stated as the above and allowed to grow for 2 weeks. The colonies were then stained with 0.1% crystal violet (Sigma-Aldrich), washed with PBS and their number was counted. Images of the colonies were scanned with a gel documentation system (AlphaImager® HP, Alpha Innotech Corporation, San Leandro, CA).

### *In vivo* tumorigenicity assay

The study protocol was approved by the Committee on the Use of Live Animals in Teaching and Research at the University of Hong Kong. BeWo cells were transfected either with 50 nM of miR-34a miRCURY LNA™ knockdown probe or control (Exiqon, Vedbaek, Denmark). The transfected BeWo cells (1 × 10^6^) were resuspended in 100 μl of PBS, mixed with 100 μl of matrigel (BD Biosciences), and injected subcutaneously into both sides of the posterior flanks of 4- to 6-week-old female B-17/Icr-scid (SCID) mice. The animals were sacrificed after 4 weeks. Four mice were used in each experiment and the experiment was repeated for 5 times independently.

### Western blot analysis

Cell lysates were prepared as described [15]. The protein expression of DLL1, NOTCH1 and β-actin were detected using specific anti-DLL1 (Santa Cruz, sc-9102), anti-NOTCH1 (Santa Cruz, sc-6014) and anti-β-actin antibodies (Santa Cruz, sc-47778). The denatured protein samples were resolved on a 8% denaturing SDS-PAGE and transferred to a nitrocellulose membrane. The membrane was blocked with Tris-buffered saline containing 5% nonfat milk and 0.5% Tween 20 (blocking buffer) at room temperature for 1 hour. Hybridization was performed at 4°C overnight (1^o^Ab 1:1000 for DLL1 and NOTCH1, 1:10000 for β-actin), followed by extensive washing and incubation with appropriate horseradish peroxidase-conjugated secondary antibody (1:2500) in blocking buffer for 1 hour at room temperature. The protein bands were detected by chemiluminescence detection.

### Immunohistochemical staining

Tissues preparation and immunohistochemistry were performed as described [16]. Briefly, antigen retrieval was performed by heating the sections in 1X target antigen retrieval solution (Dako, Glostrup, Denmark). Non-specific binding was blocked by incubating the tissue sections in PBS containing 5% serum (Sigma-Aldrich) and 0.1% Tween 20. DLL1 immunoreactivities were detected by successive incubation with specific antibody against DLL1 (Santa Cruz), biotinylated polyclonal rabbit anti-goat IgG (Dako) and Strep ABComplex/Horseradish Peroxidase HRP (Vector Laboratories, Burlingame, CA). Signal was visualized with 3,3’-diaminobenzidine (Dako).

### Statistical analysis

Each experiment was repeated independently for at least 3 times. All the values were reported as means ± SD. Differences between the treatment and the control groups were analyzed by Kruskal-Wallis test. *p* < 0.05 was considered as statistically significant.

## Results

### MiR-34a reduces proliferation and invasion of choriocarcinoma cell lines

**Figure 1:**
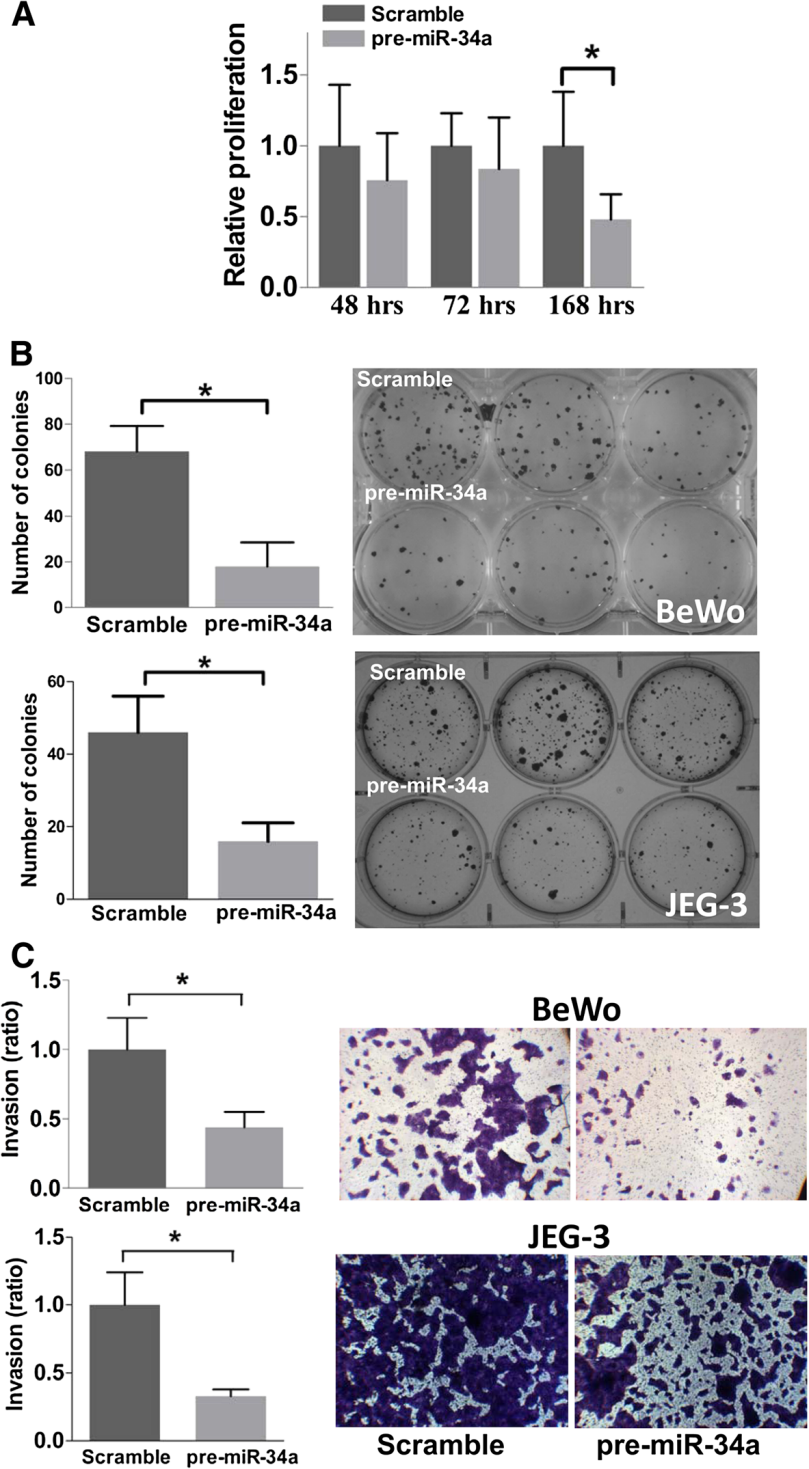
**Effect of miR-34a on choriocarcinoma cells. (A)** Cell proliferation upon miR-34a ectopic expression. Significantly slower proliferation was observed in cells with miR-34a ectopic expression at 168 hours post-transfection. **(B)** Colony formation of pre-miR-34a transfected cells seeded at low density. The colonies were visualized after staining with crystal violet at 14-days post-transfection. The bars in the chart represent mean ± SD of number of colonies from 3 independent experiments. *p < 0.05. **(C)** Suppression of invasion of BeWo and JEG-3 cells upon miR-34a ectopic expression. Representative images of the invaded cells. The graph represents the extent of invasion of the pre-miR-34a transfected cells relative to the control cells.

We first studied the biological effect of miR-34a in two choriocarcinoma cell lines BeWo and JEG-3 through transfection of pre-miR-34a. We examined the level of miR-34a in the cells at day 3 and day 10 post-transfection, and found that the pre-miR-34a transfected cells had at least ~80-fold higher levels of miR-34a than the control (Additional file 1: Figure S1). Ectopic expression of miR-34a did not significantly affect BeWo cells proliferation in the first 72-hour post-transfection (Figure 1A), but a significant reduction was observed after 7 days (168 hours) of transfection (*p* < 0.05). The colony formation ability in low seeding density was evaluated 2 weeks post-transfection and the pre-miR-34a transfected cells had around 2–3 times lower colony-forming ability than the scramble precursor transfected cells (Figure 1B, *p* < 0.05).

Next, we assessed the action of miR-34a on cell invasion. The miR-34a force-expressed cells were allowed to invade a matrigel membrane for 48 hours. It was found that the invasiveness of the miR-34a force-expressed choriocarcinoma cells was significantly decreased when compared with the control group (Figure 1C).

### Delta-like one (DLL1) is a target of miR-34a in choriocarcinoma cells

**Figure 2:**
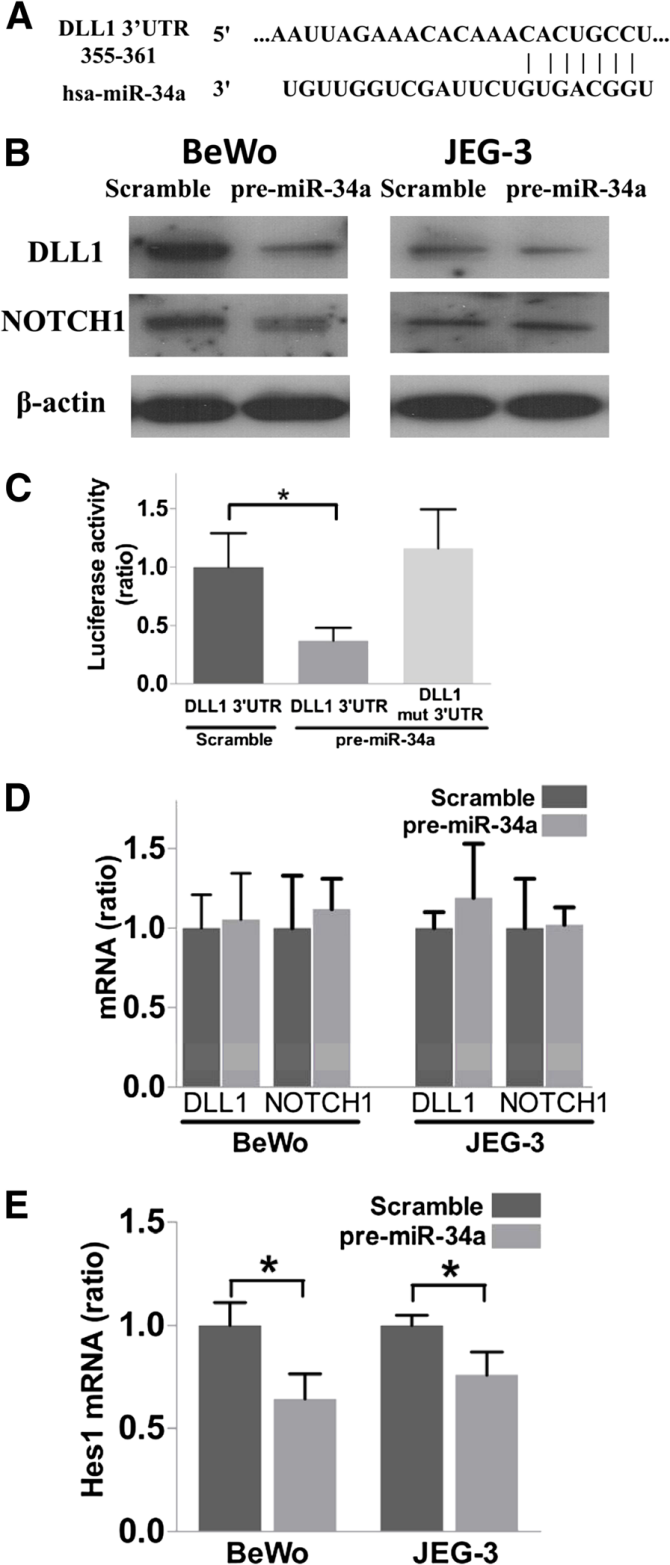
**Validation of DLL1 as a miR-34a target gene. (A)** Computational algorithm showing the seed region of miR-34a at the 3’UTR of DLL1. **(B)** Western blotting analysis of the expressions of DLL1 and NOTCH1 upon miR-34a force-expression. **(C)** Functional luciferase assay. Significant differences was found between scramble and pre-miR-34a on wild-type 3’UTR construct but not with construct carrying a mutated seed region (n = 4). **(D & E)** Quantitative real-time PCR analysis showing the mRNA levels of DLL1, NOTCH1 **(D)** and Hes-1 **(E)** between pre-miR-34a and scramble precursor transfected cells (n = 4).*p < 0.05.

Since miRNA is non-translational, it must exert its effect through regulating target genes. To determine the target gene of miR-34a, we first used *in-silico* miRNA target prediction tools to find the potential target of miR-34a. Both TargetScan 5.2 (http://www.targetscan.org/) and PictTar (http://pictar.mdc-berlin.de/) predict that the Notch ligand DLL1 is a potential target of miR-34a (Figure 2A). We examined the expression of DLL1 in BeWo and JEG-3 cells upon miR-34a force-expression for 3 days, and found that the DLL1 protein level was greatly reduced by miR-34a but not by the scramble miRNA precursor.

NOTCH1 is a known miR-34a targeted gene in choriocarcinoma cells [15]. We compared the action of miR-34a on the protein expression of NOTCH1 and DLL1. It was found that miR-34a force-expression decreased the level of DLL1 to a greater extent than that of NOTCH1 in both BeWo and JEG-3 cells (Figure 2B). Therefore, we focused our study on DLL1.

We further examined whether there is a direct interaction between miR-34a and DLL1. We constructed a luciferase reporter carrying the 3’UTR of DLL1 and transfected the reporter with either the pre-miR-34a or scramble miRNA precursor into BeWo cells. Force-expression of miR-34a reduced the luciferase reporter activity by more than 50% (*p* < 0.05, Figure 2C). To determine the specificity of the interaction, another reporter vector carrying a mutation at the putative seed binding sequence was constructed. Force-expression of miR-34a had no significant effect on the reporter activities of the mutant construct, confirming the specificity of the action of miR-34a on DLL1.

To study the mechanism of action of miR-34a on DLL1 expression, we determined the mRNA expression of DLL1 upon miR-34a force-expression, RT-qPCR revealed that the treatment and the control groups had similar levels of the DLL1 mRNA (Figure 2D). The observation indicated that miR-34a regulated DLL1 expression in choriocarcinoma cells through translational inhibition. Similarly, the expression of NOTCH1 mRNA was not affected by miR-34a force-expression. To confirm that the action of miR-34a on DLL1 modulated Notch signaling, we examined the expression of the Notch signaling target gene Hairy Enhancer of Split-1 protein (Hes-1) and found that it was reduced upon force-expression of miR-34a in both cell lines (Figure 2E).

### MiR-34a regulates invasion of BeWo cells through the Notch signaling pathway

**Figure 3:**
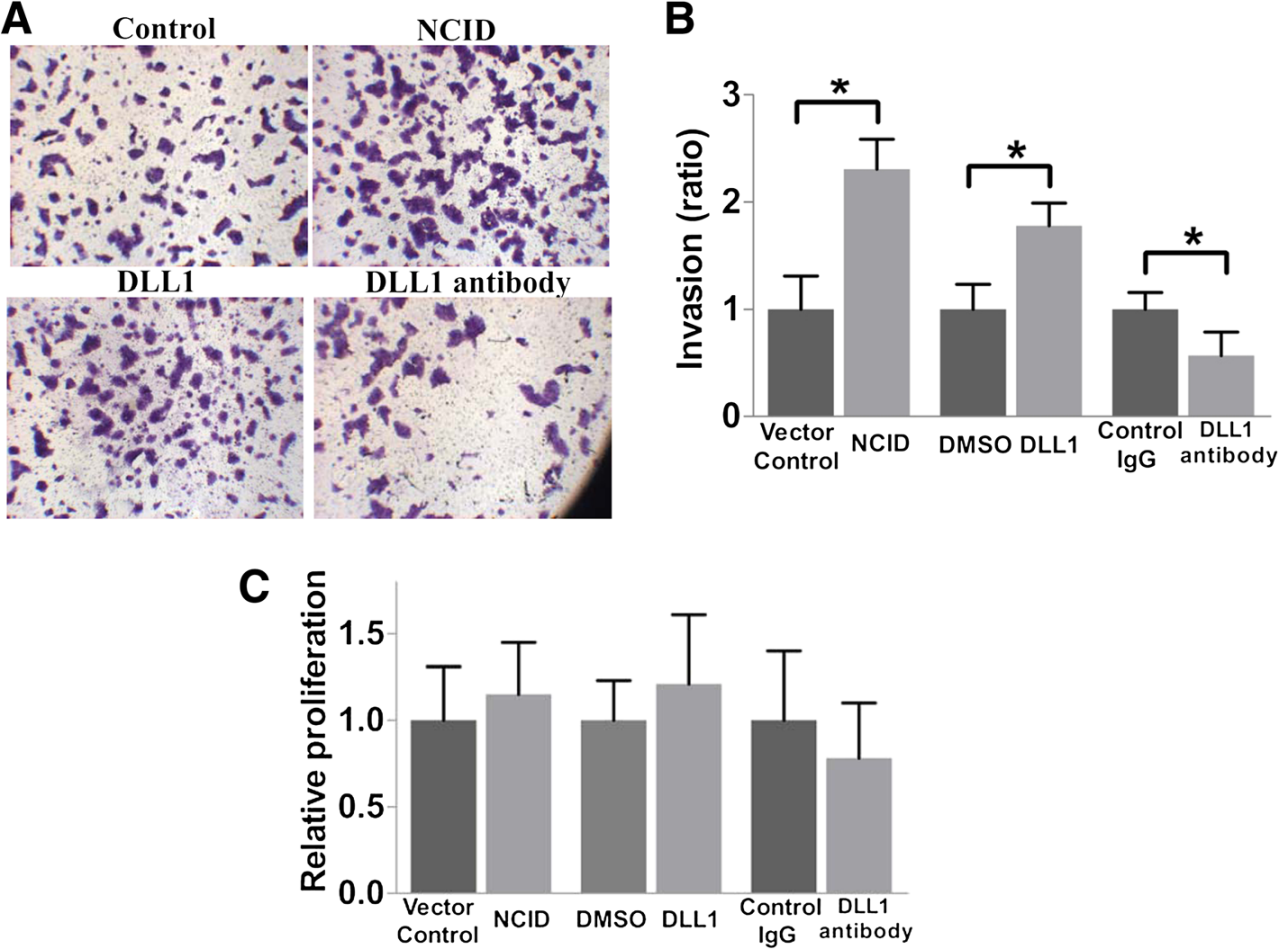
**Role of DLL1 and Notch signaling in cell invasion. (A)** Representative pictures showing increase in cell invasion after activation of Notch signaling by transfection of NCID and recombinant DLL1 treatment. The invasion of the cells was reduced by treatment with anti-DLL1 antibody. **(B)** Quantification of cell invasion relative to the untreated control cells. (n = 4). **(C)** Cell proliferation expressed as relative to the respective control cells. (n = 4).*p < 0.05.

**Figure 4:**
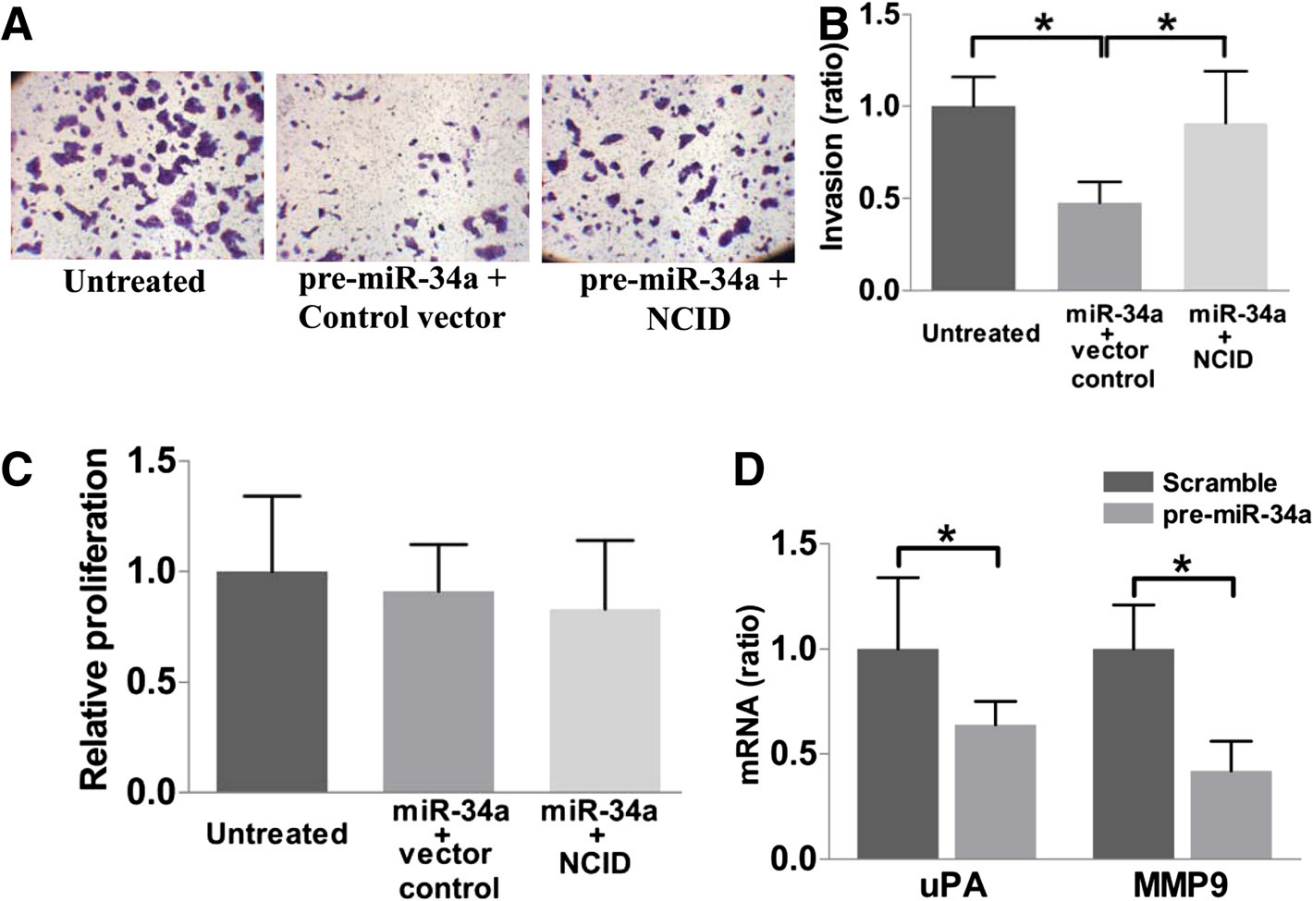
**MiR-34a reduces cell invasion through Notch signaling. (A)** Representative images showing that Notch activation nearly fully nullified the inhibitory effect of miR-34a force-expression on cell invasion. **(B)** Invasion expressed as relative to the untreated control cells (n = 4). **(C)** Proliferation of the cells. Parallel experiment demonstrated no significant effect of treatments on proliferation of the transfected cells. **(D)** uPA and MMP9 mRNA expression in the transfected cells as determined by RT-qPCRs (n = 4) *p < 0.05.

DLL1 treatment significantly increased cell invasion (Figure 3A and B), whilst treatment with anti-DLL1 antibody inhibited around 30% of the invasion. On the other hand, force-expression of NCID increased cell invasion by more than 2-fold. These treatments did not significantly affect proliferation as reflected by the cell proliferation assay (Figure 3C). We next determined the role of Notch signaling activation on the action of miR-34a on cell invasion. As shown in Figure 4A, the effect of force-expression of miR-34a was nearly completely nullified by Notch signaling activation but not by the control treatment. Again, the treatments did not affect cell proliferation (Figure 4B). Moreover, RT-qPCR showed that miR-34a force-expression reduced around 35% of the urokinase-type plasminogen activator (uPA) and 55% of the matrix metalloproteinase-9 (MMP9) expression (Figure 4C). Thus, we concluded that miR-34a force-expression reduced the invasiveness of BeWo cells through DLL1 and the Notch signaling pathway.

### MiR-34a knockdown enhances tumor growth *in vivo*

**Figure 5:**
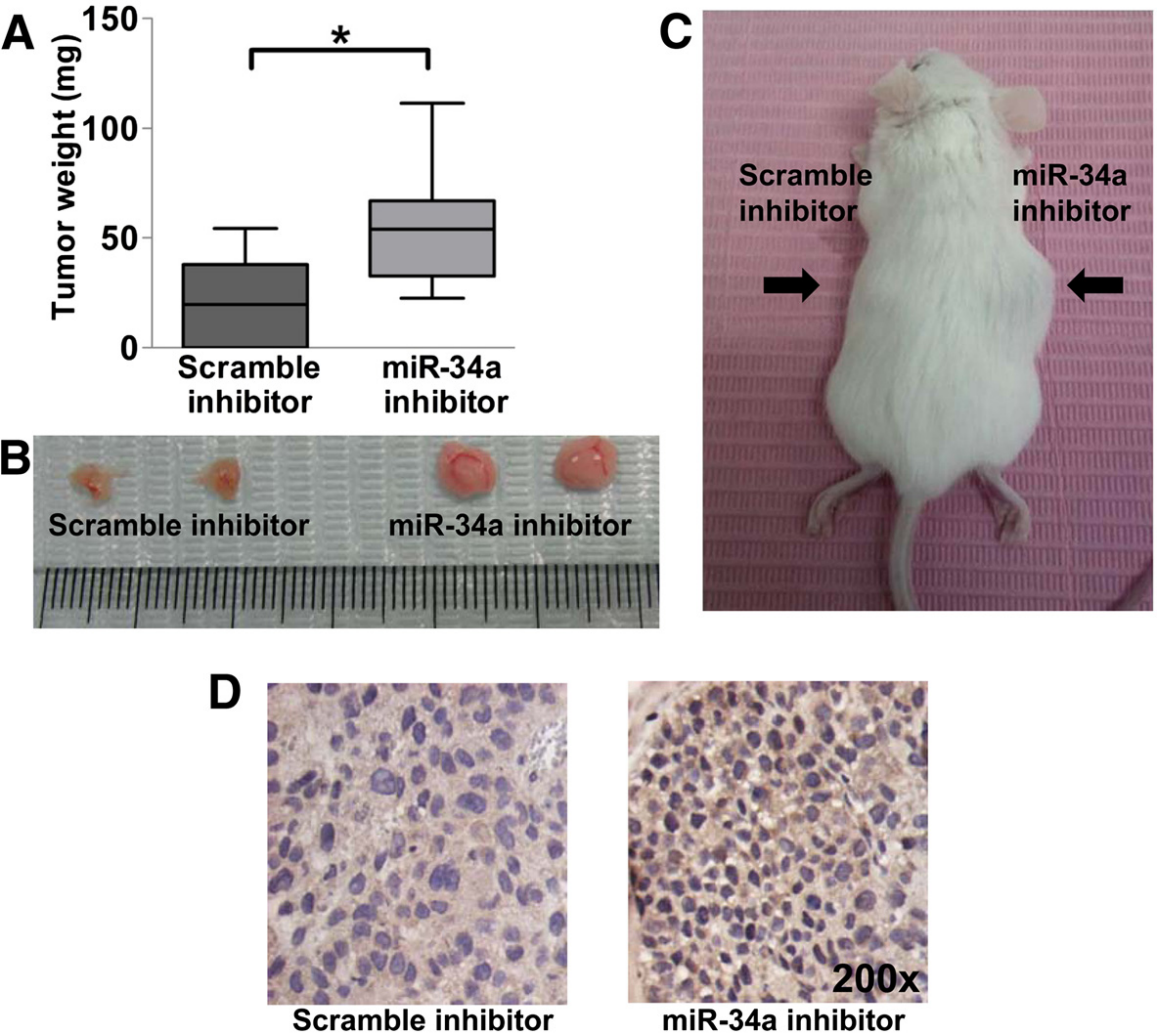
**MiR-34a inhibition enhances tumor growth *in vivo*. (A)** Weight of tumor xenografts excised from SCID mice after miR-34a knockdown. **(B)** Representative tumor xenografts excised from SCID mice. **(C)** Representative picture of SCID mice receiving subcutaneous inoculation of BeWo cells before excising for tumor xenografts. **(D)** Representative views of expression of DLL1 in xenografts upon miR-34a knockdown or scramble knockdown (Magnification: 200×).

To examine whether miR-34a knockdown affects tumor formation *in vivo*, we subcutaneously inoculated miR-34a knockdown BeWo cells or scramble knockdown cells into SCID mice. Inhibition of miR-34a significantly increased the weight of the xenografts by day 28 when compared with xenografts transfected with scramble control (*p* < 0.05, Figure 5A-C). Immunostaining showed that miR-34a knockdown increased the expression of DLL1 in the xenografts when compared to the control (Figure 5D).

## Discussion and conclusions

MiR-34 family members were first identified as tumor suppressors [10,11] and are associated with a variety of tumors [17]. However, their roles in pathogenesis are poorly understood. Recently, their family members were shown to regulate neurite outgrowth, morphology and functions [18], late steps of spermatogenesis [19] and modulate the first cleavage of mouse preimplantation embryos [20]. In this study, we explored the action of miR-34a in choriocarcinoma cell lines.

We observed a delayed action of miR-34a force-expression on proliferation, in which a significant inhibition was detected only at 168-hour post-transfection whereas a decrease in DLL1 protein level occurred at 72-hour post-transfection. Similar finding was reported in glioma stem cells [21]. DLL1 is a transmembrane ligand of the Notch signaling pathway. The delayed action could be due to the need of adequate physical contact between adjacent cells for sufficient activation of Notch signaling before an effect on proliferation could be observed, and the contact was inadequate in the early part of the experiment when the cell density was low. The explanation is consistent with a previous report demonstrating that another Notch-ligand JAG1 affects proliferation only when the cell density is above certain density [22].

p53 has a key role in inducing apoptosis and exerts its tumor-suppressive effect partially through miR-34a [10]. In many solid tumors, p53 malfunction is a consequence of gene mutation. However, direct sequencing cannot detect mutation in p53 cDNA of gestational trophoblastic disease [23,24]. In fact, p53 is highly expressed in choriocarcinoma [25] and is associated with a more aggressive behavior [26]. This is in contrast to many other cells, which undergo programmed cell death when the level of p53 is high. It is possible that there is a malfunction of the p53 effectors in the choriocarcinoma enabling the cells to survive under such condition. Suppression of the apoptosis-stimulating proteins of p53 (ASPP1), a member of the p53 transcriptional complex, through promoter hypermethylation in choriocarcinoma cell lines supports this possibility [27]. In fact, force-expression of ASPP1 in choriocarcinoma cell line has profound effects on reducing tumorigenecity [27]. The present study suggests that miR-34a is another component of the p53 network important in tumor suppression.

Metastasis is a major cause of cancer deaths while tumor invasion is an early marker of metastasis. Thus, understanding of tumor invasion is of great importance. MiR-34a inhibits invasion in a number of tumors including prostate cancer [28], colon cancer [29], cervical cancer [15] and hepatocellular cancer [30]. Our findings support these observations and further show that miR-34a regulates invasion through DLL1 leading eventually to reduction in the expression of the matrix degrading enzymes.

DLL1 is a target of miR-34a in medulloblastoma [31]. As the targets of miRNA are cell context-dependent [32], a reporter assay was conducted to confirm the direct interaction of miR-34a with DLL1 in choriocarcinoma. In fact, several Notch receptors and ligands have been demonstrated to be targets of miR-34a. These include DLL1 ([31]; this study), JAG1[15,33], NOTCH1 [15,34], NOTCH2 [34]. In this study, we also demonstrated that miR-34a inhibits NOTCH1 expression by translational inhibition in choriocarcinoma cells.

Notch signaling components are expressed in the trophoblast during normal pregnancy [35]. Apart from choriocarcinoma cell lines [15], there is no study on Notch signaling in primary choriocarcinoma tissues. In other cancers, aberrant expression and activation of Notch signaling are associated with changes in cell invasion [36]. In this study, we found that DLL1/Notch signaling mediated the action of miR-34a; activation of the Notch signaling through NCID transfection nullified the action of force-expression of miR-34a on suppressing the invasion of BeWo cells. As there are at least 3 miR-34a-targeted Notch components, DLL1, NOTCH1 and JAG1 in choriocarcinoma cells, the observed tumor suppressive effect of miR-34a and its action on Hes-1 could be a summation effect of miR-34a on these Notch targets.

Our data showed that miR-34a force-expression suppressed invasion by reducing the expression of MMP-9 and uPA. Both enzymes are regulated by AP-1 transcription factor complex [29,37]. Choriocarcinomas have a strong expression of members of the AP-1 family including, c-Jun, Jun D and Fra1 [38]. MiR-34a may regulate AP-1 complex through two pathways. The first pathway is the direct action of miRNA on its target, Fra-1 [29], which is an integral part of AP-1. One of the downstream effectors of Notch signaling is AP-1. Therefore, the second pathway is indirectly through Notch signaling. As stated above, several components of the Notch signaling are target of the miR-34 family members [15,31,33,34]. In this study, Notch signaling activation nearly fully nullified the effect of miR-34a indicating the Notch pathway being the major miR-34a target for controlling cell invasion in our model.

Notch signaling plays an important role in cancer. It is essential for cell survival and has anti-apoptotic roles [39-41]. Some tumor cells termed cancer stem cells possess stem-cell-like properties and exhibit enhanced chemoresistance and malignancy capabilities [42]. The Notch, Hedgehog and Wnt signaling pathways are the strongest stem cell promoting pathways keeping the stem cells in an undifferentiated state. It has been suggested that treatment targeting these pathways can inhibit tumor relapse and improve overall cancer survival [43,44]. For example, activation of the Notch signaling pathway can determine cancer cell stemness and tumorigenicity in certain cases [31,45]. Currently, there are evidences indicating that miR-34a is at least a suppressor of the Notch [15,33] and the Wnt signaling pathway [46], and that miR-34a force-expression negatively affects tumor-propagating cells through inhibiting DLL1 in medulloblastoma [31]. Our study provides further evidence that miR-34a reduces tumorigenicity through DLL1 but whether miR-34a regulates cancer stem cells stemness in choriocarcinoma remains to be determined.

In summary, this study demonstrates that miR-34a is a tumor suppressive miRNA in choriocarcinoma cells. The miRNA exerts its biological activities through regulation of the Notch ligand DLL1. It is possible that miR-34a can be used as a therapeutic target for treating choriocarcinoma in the future.

## Competing interests

The authors declare that they have no competing interests.

## Authors’ contributions

RTK Pang and WSB Yeung designed the experiments. RTK Pang, CON Leung, CL Lee, KKW Lam and TM Ye performed the experiments. RTK Pang and WSB Yeung analyzed the data. PCN Chiu contributed reagents/materials/analysis tools. RTK Pang and WSB Yeung wrote the paper. All authors read and approved the final manuscript.

## Pre-publication history

The pre-publication history for this paper can be accessed here:

http://www.biomedcentral.com/1471-2407/13/25/prepub

## Supplementary Material

Additional file 1 **Figure S1. **Expression level of miR-34a at different time points post-transfection. Levels of miR-34a were determined by TaqMan miRNA assays and normalized by RNU6B as described in the Materials and Methods. Relative expression level was expressed as fold over control. Click here for file
