# Supplementary material for: MicroRNA-34a is a tumor suppressor in choriocarcinoma via regulation of Delta-like1
Source: BMC Cancer. 2013 Jan 18;13:25. doi: 10.1186/1471-2407-13-25 (PMC3561246; doi:10.1186/1471-2407-13-25)
Supplement: Additional file 1 — Figure S1. Expression level of miR-34a at different time points post-transfection. Levels of miR-34a were determined by TaqMan miRNA assays and normalized by RNU6B as described in the Materials and Methods. Relative expression level was expressed as fold over control. [file 1471-2407-13-25-S1.docx]

**Supplementary Figure 1**


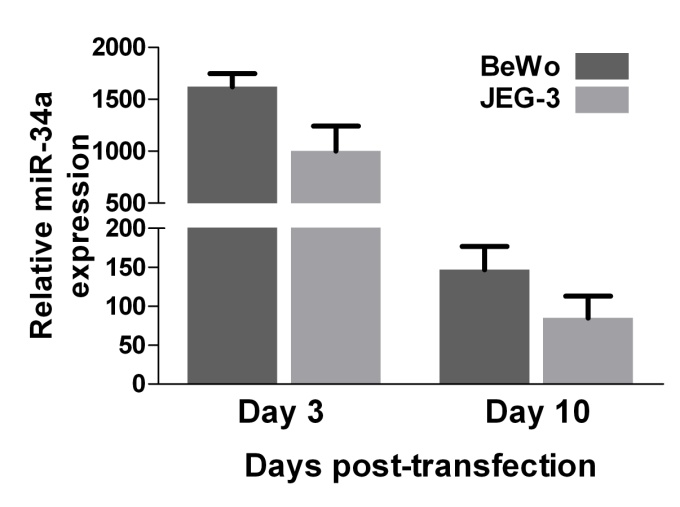


**Expression level of miR-34a at different time points post-transfection.** Levels of miR-34a were determined by TaqMan miRNA assays and normalized by RNU6B as described in the Materials and Methods. Relative expression level was expressed as fold over control.
